# Intelligent diagnosis with Chinese electronic medical records based on convolutional neural networks

**DOI:** 10.1186/s12859-019-2617-8

**Published:** 2019-02-01

**Authors:** Xiaozheng Li, Huazhen Wang, Huixin He, Jixiang Du, Jian Chen, Jinzhun Wu

**Affiliations:** 1 College of Computer Science and Technology, Huaqiao University, Xiamen, 361021 China; 2 Research Department, Zhiye software, Xiamen, 361021 China; 3 Pediatric Department, The First Affiliated Hospital of Xiamen University, Xiamen, 361003 China

**Keywords:** Chinese electronic medical records, Convolutional neural networks, Natural language processing

## Abstract

**Background:**

Benefiting from big data, powerful computation and new algorithmic techniques, we have been witnessing the renaissance of deep learning, particularly the combination of natural language processing (NLP) and deep neural networks. The advent of electronic medical records (EMRs) has not only changed the format of medical records but also helped users to obtain information faster. However, there are many challenges regarding researching directly using Chinese EMRs, such as low quality, huge quantity, imbalance, semi-structure and non-structure, particularly the high density of the Chinese language compared with English. Therefore, effective word segmentation, word representation and model architecture are the core technologies in the literature on Chinese EMRs.

**Results:**

In this paper, we propose a deep learning framework to study intelligent diagnosis using Chinese EMR data, which incorporates a convolutional neural network (CNN) into an EMR classification application. The novelty of this paper is reflected in the following: (1) We construct a pediatric medical dictionary based on Chinese EMRs. (2) Word2vec adopted in word embedding is used to achieve the semantic description of the content of Chinese EMRs. (3) A fine-tuning CNN model is constructed to feed the pediatric diagnosis with Chinese EMR data. Our results on real-world pediatric Chinese EMRs demonstrate that the average accuracy and F1-score of the CNN models are up to 81%, which indicates the effectiveness of the CNN model for the classification of EMRs. Particularly, a fine-tuning one-layer CNN performs best among all CNNs, recurrent neural network (RNN) (long short-term memory, gated recurrent unit) and CNN-RNN models, and the average accuracy and F1-score are both up to 83%.

**Conclusion:**

The CNN framework that includes word segmentation, word embedding and model training can serve as an intelligent auxiliary diagnosis tool for pediatricians. Particularly, a fine-tuning one-layer CNN performs well, which indicates that word order does not appear to have a useful effect on our Chinese EMRs.

**Electronic supplementary material:**

The online version of this article (10.1186/s12859-019-2617-8) contains supplementary material, which is available to authorized users.

## Background

### Challenges of diagnosing using EMR data

An integrated electronic medical record system is becoming an essential part of the fabric of modern healthcare, which can collect, store, display, transmit and reproduce patient information [1, 2]. The current studies show that medical information provided by Electronic Medical Records (EMRs) is more complete and faster to retrieve than traditional paper records [3]. Nowdays, EMRs are becoming the main source of medical information about patients [4]. The degree of health information sharing has become one of the indicators of hospital information construction in various countries. Therefore, the research and application of EMRs have certain scales and experiences in the world. How to use the rapidly growing EMR data to support biomedical research and clinical research is an important research content [5].

Due to their semi-structured and unstructured form, the study of EMRs belongs to the specific domain of Natural Language Processing (NLP). Notably, recent years have witnessed a surge of interests in data analytics with patient EMRs using NLP. Ananthakrishnan et al. [6] developed a robust electronic medical record–based model for classification of inflammatory bowel disease leveraging the combination of codified data and information from clinical text notes using natural language processing. Katherine et al. [7] assessed whether a classification algorithm incorporating narrative EMR data (typed physician notes) more accurately classifies subjects with rheumatoid arthritis (RA) compared with an algorithm using codified EMR data alone. The work by Ruben et al. [8] studied a real-time electronic predictive model that identifies hospitalized heart failure (HF) patients at high risk for readmission or death, which may be valuable to clinicians and hospitals who care for these patients. Although some effective NLP methods have been proposed for EMRs, lots of challenges still remain, to list a few among the most relevant ones:

(1) *Low-Quality.* Owing to the constraint of electronic medical record template, the EMRs data are similar in a large scale, especially the content of EMRs. What’s more, the medical records writing is not standardized which sometimes shows inconsistency between records and doctor’s diagnosis.

(2) *Huge-Quantity.* With the increasing popularity of medical information construction, EMRs data have been growing rapidly in scale and species. There is a great intensive knowledge to explore in the EMRs databases.

(3) *Imbalance.* Due to the wide variety of diseases (e.g., there are more than 14,000 different diagnosis codes in terms of International Classification of Diseases - 9th Version (ICD-9)) in EMRs data, the sample distribution is expected to remain rather imbalance.

(4) *Semi-structure and non-structure.* The EMRs data include front sheet, progress notes, test results, medical orders, surgical records, nursing records and so on. These documents include structured information, unstructured texts and graphic image information.

Despite the above challenges, one must address the additional challenges posed by the high density of the Chinese language compared with other languages [9]. Most of words in Chinese corpus cannot be expressed independently. Therefore, the word segmentation is a necessary preprocessing step, and its effect directly affects the following series NLP operations for EMRs [10].

### Intelligent diagnosis using EMR data

In practice, a great deal of information is used to determine the disease, such as the patient’s chief complaint, current history, past history, relevant examinations. However, the diagnostic accuracy not only depends on individual medical knowledge but also clinical experience. Different doctors may have different diagnoses on the same patient. In particular, doctors with poor skills or in remote areas have lower diagnostic accuracy. Therefore, it’s very important and realistic to establish a intelligent dignosis model for EMRs.

Chen et al. [11] applied machine learning methods, including support vector machine (SVM), decision forest, and a novel summed similarity measure to automatically classify the breast cancer texts on their Semantic Space models. Ekong et al. [12] proposed the use of fuzzy clustering algorithm for a clinical study on liver dysfunction symptoms. Xu et al. [13] designed and implemented a medical information text classification system based on a KNN. Many researchers at home and abroad, who use EMRs for disease prediction, always focus on a particular department as well as a specific disease. At present, the algorithms used by researchers mostly focus on machine learning methods, such as KNN, SVM, DT. Due to the particularity of medical field and the key role of professional medical knowledge, common text classification methods often fail to achieve good classification performance and cannot meet the requirement of clinical practice [14].

Benefiting from big data, powerful computation and new algorithmic techniques, we have been witnessing the renaissance of deep learning, especially the combination of natural language processing and deep neural networks. Dong et al. [15] presented a CNN based multiclass classification method for mining named entities with EMRs. A transfer bi-directional Recurrent Neural Networks was proposed for named entity recognition (NER) in Chinese EMRs that aims to extract medical knowledge such as phrases recording diseases and treatments automatically [16]. SA [17] marked the prediction of heart disease as a multi-level problem of different features or signs and constructed an IHDPS (Intelligent Heart Disease Prediction System) based on neural networks.

However, to the best of our knowledge, few significant models based on deep learning have been employed for the intelligent diagnosis with Chinese EMRs. Rajkomar et al. [18] demonstrated that deep learning methods outperformed state-of-art traditional predictive models in all cases with electronic health record (EHR) data, which is probably the first research on using deep learning methods in EHR model analysis.

### Deep learning for natural language processing

NLP is a theory-motivated range of computational techniques for the automatic analysis and representation of human language, which enables computers to perform a variety of natural language related tasks at all levels, ranging from parsing and part-of-speech (POS) tagging, to dialog systems and machine translation. In recent years, Deep learning algorithms and architectures have already won numerous contests in fields such as computer vision and pattern recognition. Following this trend, recent NLP research is now increasingly focusing on the use of deep learning methods [19].

In a deep learning with NLP model, word embedding is usually used as the first data preprocessing layer. It’s because the learnt word vectors can capture general semantic and syntactical information, that word embedding produces state-of-art results on various NLP tasks [20–22]. Following the success of word embedding [23, 24], CNNs turned out to be the natural choice in view of their effectiveness in computer vision and pattern recognition tasks [25–27]. In 2014, Kim [28] explored using the CNNs for various sentence classification tasks, and CNNs was quickly adapted by some researchers due to its simple and effective network. Poria et al. [29] proposed a multi-level deep CNN to tag each word in a sentence, which coupled with a group of linguistic patterns and finally performed well in aspect detection.

Besides text classification, CNN models are also suitable for other NLP tasks. For example, Denil et al. [30] applied DCNN to map meanings of words that constitute a sentence to that of documents for summarization, which provided insights in automatic summarization of texts and the learning process. In the domain of Question and Answer (QA), the work by Yih et al. [31] presented a CNN architecture to measure the semantic similarity between a question and entries in a knowledge base (KB), which determined what supporting fact in the KB to look for when answering a question. In the domain of Information and Retrieval (IR), Chen et al. [32] proposed a dynamic multi-pooling CNN (DMCNN) strategy to overcome the loss of information for multiple-event modeling. In the speech recognition, Palaz et al. [33] performed extensive analysis based on a speech recognition systems with CNN framework and finally created a robust automatic speech recognition system. In general, CNNs are extremely effective in mining semantic clues in contextual windows.

It is well known that pediatric patients are generally depauperate, traversing from newborns to adolescents. Correspondingly, the treatment and dosage of medicine are different from those given to adult patients. Thus, it is a great challenge to build a prediction model for pediatric diagnosis that is trained to “learn” expert medical knowledge to simulate the doctor’s thinking and diagnostic reasoning.

In this research, we propose a deep learning framework to study intelligent diagnosis using Chinese EMRs, which incorporates a convolutional neural network (CNN) into an EMR classification application. This framework involves a series of operations that includes word segmentation, word embedding and model training. In real pediatric Chinese EMR intelligent diagnosis applications, the proposed model has high accuracy and a high F1-score, and achieves good results. The novelty of this paper is reflected in the following:

(1) We construct a pediatric medical dictionary based on Chinese EMRs.

(2) Word2vec is used as a word embedding method to achieve the semantic description of the content of Chinese EMRs.

(3) A fine-tuning CNN model is constructed to feed the pediatric diagnosis with Chinese EMR data.

## Methods

### Proposed framework

**Fig. 1:**
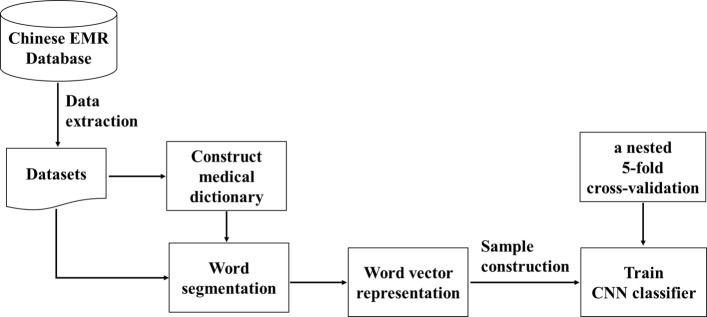
Schema of our proposed framework. NLP technology involves a series of operations, which includes word segmentation, word embedding and model training

Our proposed framework is the incorporation of a CNN into the procedure of NLP with Chinese EMRs, and its schema is shown in Fig. 1, which includes word segmentation, word embedding and model training. First, the corpus is extracted from the Chinese EMR database. Then, a medical dictionary is constructed from the original corpus, which is used as external expert knowledge in word segmentation. Next, word embedding is executed. Finally, the CNN model is trained using a nested 5-fold cross-validation approach. The detailed design of our proposed framework is presented in the following.

### Datasets

In this paper, we explore our proposed framework for pediatric Chinese EMRs. A total of 144,170 valid medical records were collected, which includes 63 types of pediatric diseases.

The number of samples that are “acute upper respiratory tract infection” accounts for more than 50%; hence, the sample distribution with 63 types of pediatric diseases is rather imbalanced. To reduce the effect of the unbalanced dataset on the prediction model, three types of smaller datasets were constructed by downsampling the data to explore the effectiveness of our proposed framework: eight types of diseases with large sample sizes and a great difference in diseases; the top 32 types of diseases sorted by sample size; and seven types of diseases excluding "acute upper respiratory tract infection". Therefore, the text classification of 7, 8, 32 and 63 diseases were studied separately to explore the universality of the CNN model for the intelligent diagnosis of pediatric outpatients. The distribution of the experimental datasets is given in Table 1.

**Table 1:** Distribution of datasets with respect to four types of classification applications for pediatric Chinese EMRs

Number of diseases	Name of diseases	Number of samples
7	Allergic rhinitis, bronchitis, acute bronchitis, respiratory disease, bronchial asthma, no critical, diarrhea, cough variant asthma	49,148
8	**acute upper respiratory tract infection**, allergic rhinitis, bronchitis, acute bronchitis, respiratory disease, bronchialasthma, no critical, diarrhea, cough variant asthma	92,744
32	See Additional file 1	132,637
63	See Additional file 1	144,170

Boldface represents an additional disease compared with the seven-classification application

### Word segmentation

Word segmentation refers to word sequences that are divided into the smallest semantically independent expressions using an algorithm [34]. Generally, there are four types of mainstream methods: dictionary-based, statistics-based, comprehension-based and AI-based. Dictionary-based word segmentation is widely used because of its maturity and easy implementation [35]. In the process of Chinese word segmentation, particularly in specific fields such as medicine, the completeness and accuracy of domain dictionaries largely determine the performance of the word segmentation system [34]. For example, when “upper respiratory tract infection” is the official, full name of the disease, some Chinese physicians write “upper infection” as an informal abbreviation [36].Establishing a fast, accurate and efficient word segmentation dictionary fundamentally affects the performance of word segmentation.

**Fig. 2:**
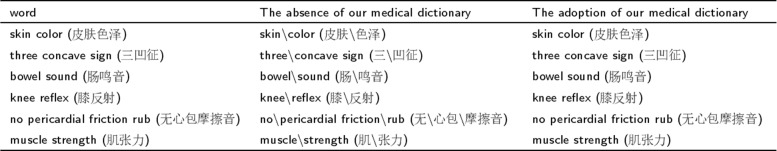
Semantic rationality of whether to use our medical dictionary

To the best of our knowledge, there are few medical dictionaries published about pediatrics. To improve the accuracy of word segmentation, a pediatric medical dictionary with a scale of 900 was established based on the collected EMR data, which was used as expert knowledge. The public jieba word segmentation system was used, with a precise pattern, and the results are shown in Fig. 2.

### Word vector representation

The core issue of NLP is how to convert a corpus into vectors; that is, each word needs to be embedded into a mathematical space to obtain the word vector expression. There are two types of mainstream methods: one-hot and word2vec. One-hot is an intuitive expression that represents each word as an *N*-dimensional vector of the same size as the vocabulary. Generally, the value of the attribute that corresponds to the word is one and the values of other attributes are zero. With a vocabulary scale of 5850 for the seven-classification dataset, the word “cough” is expressed as [0 0 0 **1** 0 0 0 0 0 0 0 0 0 0 0 0 ]_5850_ and the word “fever” is expressed as [0 0 0 0 0 0 0 0 **1** 0 0 0 0 0 0 0 ]_5850_. However, there are some defects in this method, such as the “dimensionality disaster” and semantic gap.

Therefore, word2vec was developed to map words to obtain K-dimensional vectors; that is, word2vec uses a low-dimensional vector to represent a large amount of potential information of a word, which overcomes the “dimensionality disaster” phenomenon. Additionally, the similarity of vectors can reflect their semantic similarity [37]. Word2vec is widely used in NLP, such as word clustering, POS-tagging, syntactic analysis and emotional analysis. In the application of word2vec, it can be divided into the CBOW model and skip-gram model. The CBOW model predicts the current word using its context word and the skip-gram model predicts its context using the current word [38]. In the training procedure, the hierarchical softmax algorithm, negative sampling algorithm and sub-sampling technology were used [24, 39–43].

In our study, the CBOW strategy was adopted, with the word frequency threshold set to 5 (i.e., the least number of words that appear in the corpus), and the window size set to 5 (i.e., the number of words in the context). When determining the dimension of word vectors, Mikolov et al. [24] suggested that the classification applications of different scales should have different embedding dimensions. Therefore, the four types of text classification applications in this paper have 50, 80, 100 and 100 embedding dimensions, respectively, based on their accuracies with an optimal one-layer CNN. The relationship between accuracy and dimension is shown in Table 2.

**Table 2:** One-layer CNN accuracy for different dimensions with respect to four types of classification applications

Text classification	50 (%)	80 (%)	100 (%)
7 classes	**83.72**	83.65	83.63
8 classes	82.26	**82.55**	82.51
32 classes	73.13	73.44	**73.54**
63 classes	70.39	71.06	**71.2**

Boldface represents the best

Consider the seven-classification application as an example. Each word is embedded into 50-dimensional vector space. For instance, the word “cough” is expressed as [-3.982, -0.670, -1.754,, 3.048]_50_ and the word "fever" is expressed as [-4.487, -5.976, -5.417,, 1.216]_50_. Additionally, the word vector representation using word2vec can use the cosine distance to measure the degree of semantic similarity [10]. The cosine distance of words between “cough” are given in Table 3, which indicates that the smaller the cosine value, the more similar the semantics.

**Table 3:** Semantic similarity of word vectors

Word	Cosine distance
Recurrent cough	0.6350
Quiet cough	0.6196
Bad cough	0.5433
Little cough	0.5204
Dry cough	0.5208
Nasal obstruction	0.5914
Phlegm	0.5434
Vomiting	29.48
Afternoon	23.41
Muscular stiffness	22.83

### Convolutional neural networks

**Fig. 3:**
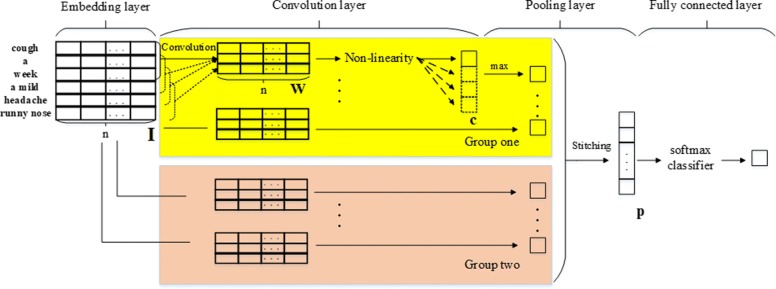
Structure of a CNN. Different from the traditional feed-forward neural network, a CNN is a multi-layer neural network, which includes four parts: embedding layer, convolution layer, pooling layer and fully connected layer

CNNs proposed by Lecun in 1989 [44] enable automatic feature representation learning. Different from the traditional feed-forward neural network, a CNN is a multi-layer neural network that includes four parts, embedding layer, convolution layer, pooling layer and fully connected layer, as illustrated in Fig. 3 [45].

The first layer is the input layer, which is an embedding matrix ${\boldsymbol {I}} \in \mathbb {R}^{{S* N}}$ that corresponds to the symptom text to be classified. Number of rows *S* is the number of words in the sentence and number of columns *N* is the dimension of the word vector. Consider the description of “cough for a week, a mild headache and runny nose" as an example. The sentence is divided into "cough + a + week + a mild + headache + runny nose” when the dictionary-based word segmentation method is used. Then each word is converted into a vector using word2vec, subsequently forming embedding matrix ***I*** as the input layer of the CNN [45].

Then different filters are applied to different layers and the result is downsampled using the pooling layer. CNNs realize automatic feature representation learning through multiple layers of networks, the core of which lies in the convolutional layer and pooling layer. The convolution layer extracts local features, whereas the pooling layer reduces the dimension of the structured feature [46, 47].

Additionally, the depth of neural networks plays a decisive role in the performance of a CNN model, and is regarded as one of the most investigated approaches used to increase its accuracy. For instance, Wang et al. [48] discussed the influence of the varied depth on the validation set of ILSVRC and proposed that “going deeper” is an effective and competitive approach to increase the accuracy of classification. The work by Hussam et al. [49] proposed a deep neural network comprised of 16 convolutional layers compressed with the Fire module adapted from the SqueezeNet model.

### Hyperparameter setup

The architecture of CNN needs fine-tuning to obtain optimal performance on specific datasets. Generally, hyperparameter setup refers to the grid-search of several parameters, which include size of filter windows, number of feature maps, dropout rate, activation function, mini-batch size, and so on [28]. Practically, the hyperparameter setup of CNN refers the filter windows of 7, 6, 5, 4 and 3, the feature maps of 128, 100, 64, 50, 32 and 16, the mini-batch size of 100, 95, 64, 50 and 32. In our experiments, a nested 5-fold cross-validation approach was applied on the seven-classification dataset, where the inner cross-validation was used for the grid-search to tune the hyperparameters, and the outer cross-validation was adopted to evaluate the performance of different models mentioned in this paper. As a result, we found that the one-layer CNN outperformed on the EMR-based pediatric diagnosis, whose hyperparameters included the filter windows of 7, the feature maps of 100, the dropout rate of 0.5, activation of relu and mini-batch size of 64, and the update rule of AdaMax. All the experiments were conducted using Python 3.5 with Python packages.

## Results

### Evaluation

In this paper, we study the effectiveness of our proposed framework on real-world pediatric Chinese EMR data. For each dataset, three metrics were used to evaluate the effectiveness and performance of algorithms: accuracy, precision and F1-score. Precision and recall were often combined to obtain a better understanding of the performance of the classifier. Their formulas for calculation are as follows:

1 $$\begin{array}{@{}rcl@{}} Accuracy = \frac{TP + TN}{TP + FP + TN + FN} \end{array} $$

2 $$\begin{array}{@{}rcl@{}} Precision = \frac{TP}{TP + FP} \end{array} $$

3 $$\begin{array}{@{}rcl@{}} Recall = \frac{TP}{TP + FN} \end{array} $$

4 $$\begin{array}{@{}rcl@{}} F1-score = \frac{2*Precision*Recall}{Precision + Recall} \end{array} $$

where

true positive (TP): scenario in text classification in which the classifier correctly classifies a positive test case into a positive class;

true negative (TN): scenario in text classification in which the classifier correctly classifies a negative test case into a negative class;

false positive (FP): scenario in text classification in which the classifier incorrectly classifies a negative test case into a positive class;

false negative (FN): scenario in text classification in which the classifier incorrectly classifies a positive test case into a negative class.

### Performance of the CNN models

In the CNN experiments, we focused on the impact of depth on our application, that is, three different depths, depth 1, depth 2 and depth 3, were explored to obtain an optimal solution. Subsequently, the comparative results with respect to the seven-classification application are presented in Table 4, which contains the precision, accuracy and F1-score of each fold.

**Table 4:** Comparative results of the CNN model with the seven-classification application

Depth	One-layer CNN(%)			Two-layer CNN(%)			Three-layer CNN(%)		
Fold ∖metrics	Precision	Accuracy	F1-score	Precision	Accuracy	F1-score	Precision	Accuracy	F1-score
1	84.26	84.1	84.16	83.13	82.9	82.97	83.05	82.74	82.84
2	83.63	83.5	83.52	82.65	82.42	82.5	82.32	81.53	81.66
3	83.86	83.55	83.61	82.54	82.26	82.35	79.09	78.89	78.94
4	84.07	83.75	83.84	82.78	82.51	82.58	82.28	82.02	82.05
5	83.87	83.71	83.76	82.97	82.81	82.85	82.6	82.37	82.4
Average	83.94	83.72	83.78	82.81	82.58	82.65	81.87	81.51	81.58

**Fig. 4:**
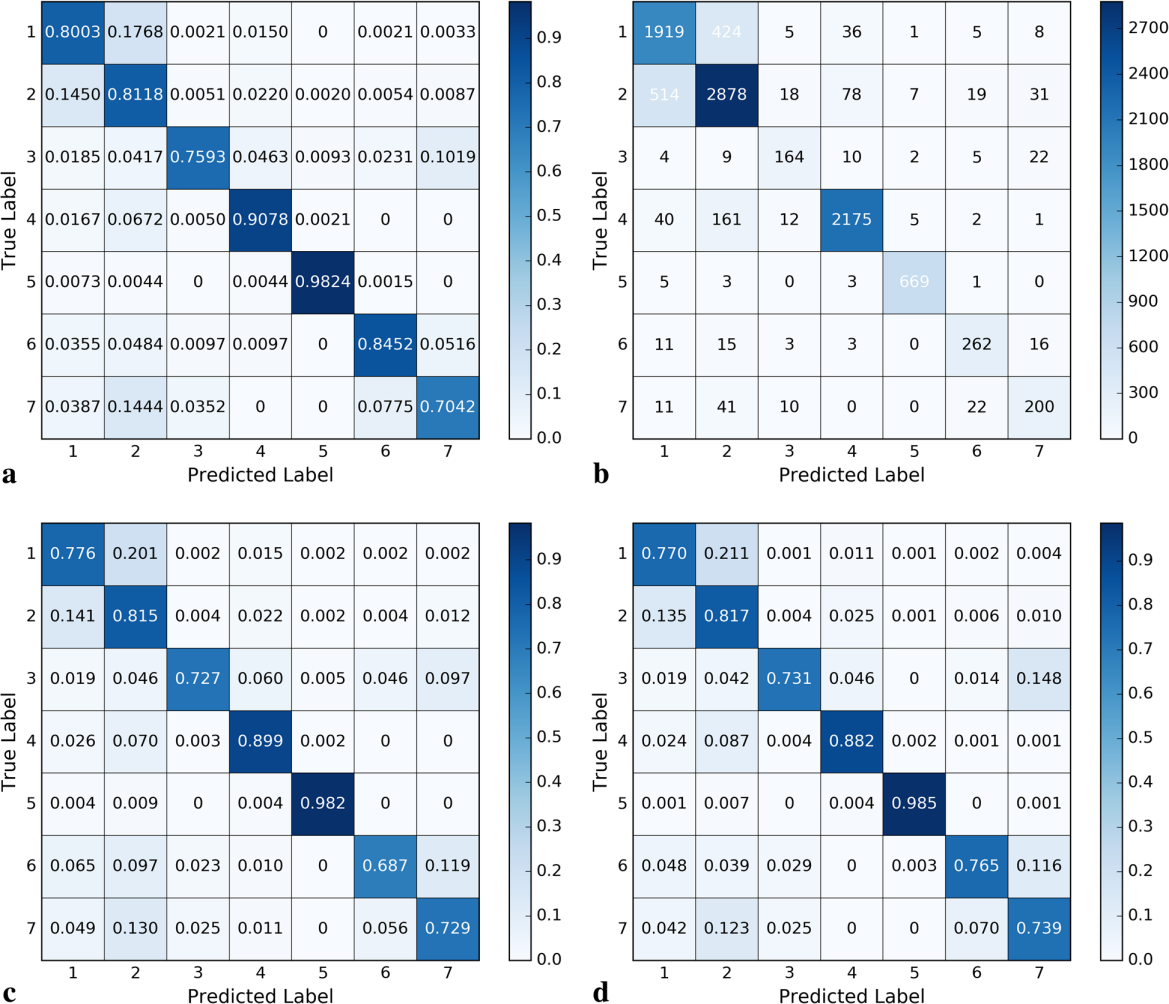
Confusion matrix of the three CNN models. **a** normalized confusion matrix of one-layer CNN. **b** unnormalized confusion matrix of one-layer CNN. **c** normalized confusion matrix of two-layer CNN. **d** normalized confusion matrix of three-layer CNN

It can be seen from Table 4 that the accuracies of the three CNN models were all higher than 81%, and the same is true for other metrics. This result indicates the effectiveness of CNN for the classification of Chinese EMRs. Furthermore, one-layer CNN had the best performance among all the CNN models, which makes it the most practicable tool in pediatric diagnosis. Because the experimental datasets were more than two classes and imbalanced, the confusion matrix of the three CNN models are shown in Fig. 4, where Fig. 4a and b show the first-fold normalized confusion matrix and its non-normalized confusion matrix for the one-layer CNN model in the outer 5-fold cross-validation, respectively. The first-fold normalized confusion matrix of the two-layer CNN model and three-layer CNN model can be observed in Fig. 4c and d, respectively.

### CNN vs. RNN models

The results of our CNN models against other methods are presented in Table 5. The model of long short-term memory (LSTM) did not perform well. The average accuracy and F1-score of the CNN models are up to 81%, which indicates the effectiveness of the CNN model for the classification of EMRs. Particularly, a fine-tuning one-layer CNN performs best among all CNN, recurrent neural network (RNN) (LSTM, gated recurrent unit (GRU)) and CNN-RNN models, and the average accuracy and F1-score are both up to 83%.

**Table 5:** Results of our CNN models against other methods

Model	Precision(%)	Accuracy(%)	F1-score(%)
1-layer CNN	**83.94**	**83.72**	**83.78**
1-layer LSTM	43.97	46.33	38.18
1-layer GRU	82.95	82.2	82.37
2-layers CNN	82.81	82.58	82.65
2-layers LSTM	23.01	34.12	19.57
2-layers GRU	83.03	82.4	82.57
3-layers CNN	81.87	81.51	81.58
CNN-1LSTM	83.86	83.55	83.62
CNN-2LSTM	83.63	83.18	83.33
CNN-1GRU	83.42	83.02	83.13
CNN-2GRU	83.52	82.95	83.1

Boldface represents the best

Based on the best CNN model architecture (one-layer CNN), the other classificaion applications, i.e., eight-classification application, 32-classification application, and 63-classification application, were evaluated by the 5-fold cross-validation. Table 6 shows the model accuracies of four types of pediatric diagnosis applications. It can be seen that (1) the highest accuracy was exhibited in the seven-classification application, which may have been caused by the small scale and somewhat balanced distribution of sample data; and (2) with the increase of disease types, the accuracy of the one-layer CNN model decreased. The main reason was that, because of the constraint of the EMR template, the content of the EMRs were similar on a large scale. Furthermore, there were not sufficient samples to train for so many different types of diseases.

**Table 6:** Accuracies of fine-tuning the one-layer CNN model with respect to four types of classification applications

The number of diseases	precision(%)	accuracy(%)	F1-score(%)
7 classes	**83.94**	**83.72**	**83.78**
8 classes	82.35	82.55	82.27
32 classes	73.09	73.54	72.5
63 classes	70.59	71.2	69.61

Boldface represents the best

## Discussion

### Impact of the Chinese medical dictionary on word segmentation

With the dictionary-based word segmentation method incorporating our pediatric medical dictionary, the corpus can be separated by " ∖". Fig. 2 shows the semantic rationality of whether to use our medical dictionary. The second column shows the segmentation result with the absence of our medical dictionary and the third column shows the segmentation result with the adoption of our medical dictionary. This shows that adopting the medical dictionary as expert knowledge accurately divided the corpus into the smallest semantic independent medical expressions, which was very helpful for the subsequent model construction.

### Impact of various example constructions

**Fig. 5:**
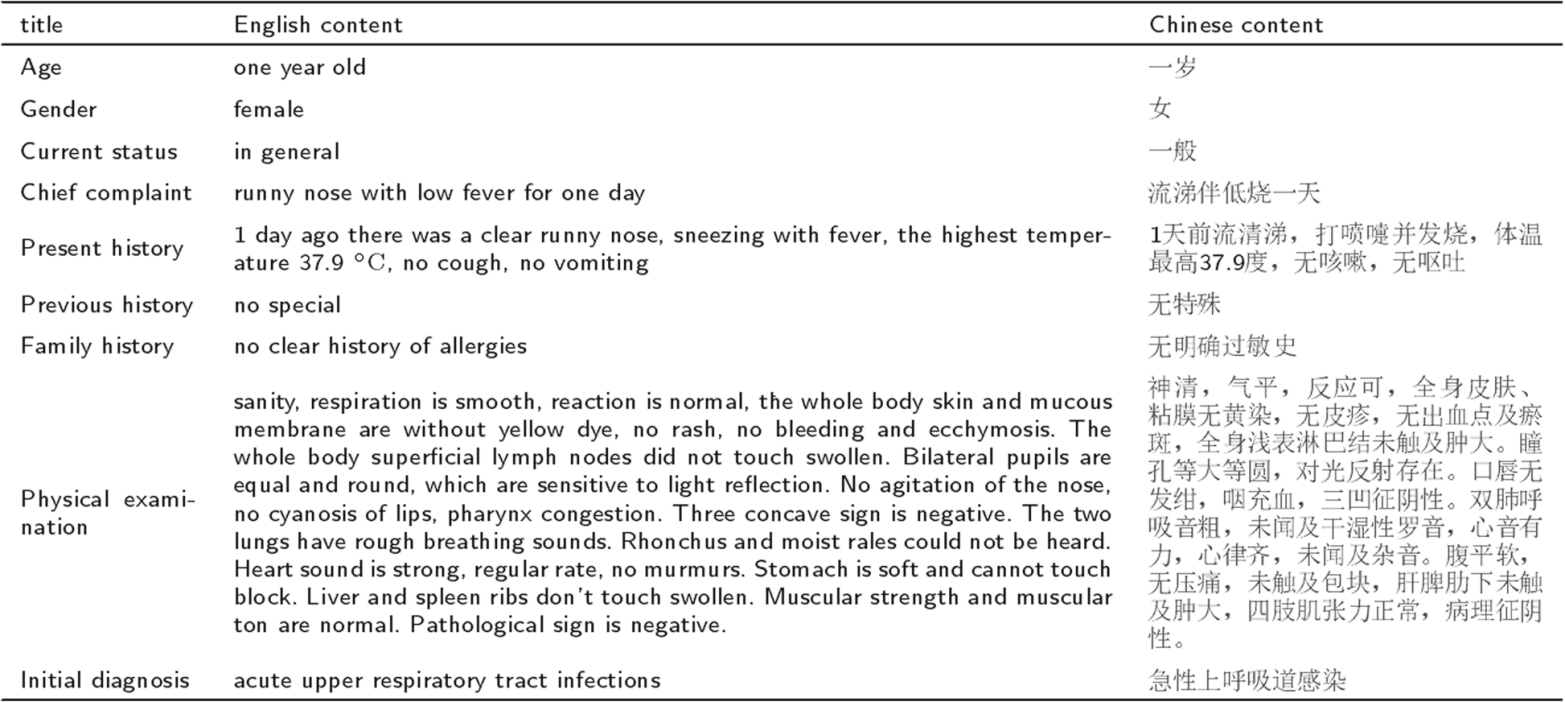
Description of a typical pediatric Chinese EMR datum

A typical medical record always contains a set of entries, such as *age, gender, current status, chief complaint, present history, previous history, family history, physical examination and diagnosis*. An example of a medical record from the pediatric Chinese EMRs is shown in Fig. 5.

Based on Fig. 5, the entry of *age, gender, current status, chief complaint, present history, previous history, family history and physical examination* are designated as the corpus, and *the initial diagnosis* is designated as the label.

When applying a CNN model, it is necessary to convert a medical record corpus into a fixed-size matrix. Considering the seven-classification application as an example, the corpus shown in Fig. 5 should be converted into a 120 ×50 matrix for training, and the number of words in each corpus is regularized to be 120 and the vector dimension of each word is 50. However, because the length of different medical records is different, that is, the number of words in the shortest corpus is 21 and the number of words in the longest corpus is 271, a corpus that contains records of various lengths should be truncated or filled to make the records even. If the shortest medical record is chosen as the regularized length, then important information in a longer corpus may be truncated. Conversely, choosing the length of the longest medical record can add too many unwanted messages (fill 0) to a shorter corpus, and increase the complex of model training.

Therefore, we attempted to explore how three types of setup, that is, a regularized length of corpus, the truncation approach and the filling mode of the medical record, affect the performance of the CNN model. For the parameter of a regularized length, we attempted 90, 100, 110, 120, 130 and 140; for the parameter of the filling mode, we considered two alternatives, that is, head-filling and tail-filling; and for the parameter of the truncation approach, we also considered two candidates, that is, head-truncation and tail-truncation. Thus, a grid-search method was adopted to determine an optimal parameter setup for the aforementioned best performing CNN model (one-layer CNN).

**Fig. 6:**
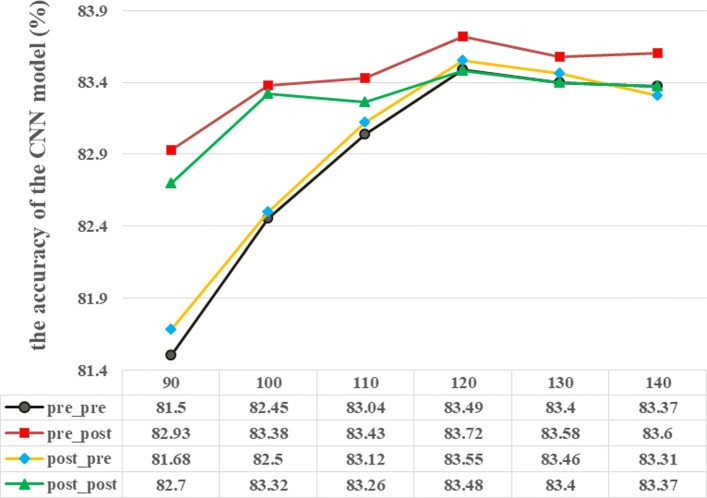
Impact of three types of parameter on the accuracy of the CNN model. Note: “pre” refers to head-filling or head-truncation and “post” refers to tail-filling or tail-truncation. For example, “pre_post” means that short text is filled by head and long text is truncated by tail

Because of the limited length of this paper, the performance of the seven-classification CNN model is illustrated in Fig. 6. The results of other classification applications were similar to those of Fig. 6. From Fig. 6, we can see that the model had very robust superiority for the configuration that had the corpus length of 120, in addition to using head-filling for shorter text and tail-truncation for the longer text, which indicates that head information for longer medical records is more important than tail information, and head-filling for shorter medical records is better than tail-filling. Therefore, for this optimal configuration, that is, where the regularized length of the corpus is 120, a head-filling mode and a tail-truncation approach for the medical record were adopted in our application.

### Impact of the class weights in training

In order to improve the class accuracy of small-number class caused by the unbalance distribution, different class weights serves as error-recognition penalty were introduced.

5 $$\begin{array}{@{}rcl@{}} class\_weights = \frac{n\_samples}{n\_classes * n\_class\_samples} \end{array} $$

where *n_samples* is the number of samples, *n_classes* is the class number of samples and *n_class_samples* is the sample number of one class.

Based on the best CNN model architecture (one-layer CNN), Table 7 shows the comparative accuracies of each class with respect to the seven-classication application and the eight-classication application, and Table 8 shows the three model evaluation indices. It can be seen that: (1) the class accuracy of small number of samples has promots a lot when using class weights, at the same time, the class accuracy of large sample size has put down a lot; and (2) In a comprehensive view, it performs well in all three metrics than using the class weights. Therefore, we do not use class weights in our article.

**Table 7:** Comparative accuracies with respect to the seven-classication application and the eight-classication application of whether to use class weights

Class ∖metrics	Name of class	Sample size	Seven-classication		Eight-classication	
			Without class weight	With class weight	Without class weight	With class weight
Class1	Allergic rhinitis	1079	71.09	**80.1**	59.68	**77.85**
Class2	Respiratory disease	11980	**90.37**	87.92	85.28	**86.3**
Class3	Cough variant asthma	1418	70.31	**80.74**	67.12	**81.45**
Class4	Acute bronchitis	11990	77.5	**80.00**	65.56	**81.78**
Class5	Bronchialasthma, no critical	1550	79.23	**83.56**	78.82	**80.77**
Class6	Bronchitis	17726	**82.79**	73.42	**66.94**	51.42
Class7	Diarrhea	3405	97.91	**98.7**	94.9	**97.06**
Class8	Acute upper respiratory tract infection	43596	NA	NA	**92.94**	84.11

Boldface represents the best

**Table 8:** Comparative results with respect to the seven-classication application and the eight-classication application of whether to use different class weights

Metrics	Seven-classication		Eight-classication	
	Without class weight	With class weight	Without class weight	With class weight
Precision (%)	**83.94**	82.27	**82.35**	80.97
Accuracy (%)	**83.72**	80.99	**82.55**	78.15
F1-score (%)	**83.78**	81.25	**82.27**	78.45

Boldface represents the best

## Conclusions

Considering the advantage of CNNs in local feature extraction and modeling performance, we attempted to explore a framework based on a CNN model for intelligent diagnosis with pediatric Chinese EMRs. Our framework was composed of three parts: word segmentation, word embedding and model training. With an expert dictionary based on collected Chinese EMR data used in word segmentation, and the word vector representation of the medical records using word2vec, we validated the effectiveness of our proposed framework on real-world EMR data. A wide range of models, which included CNN models, RNN models (LSTM, GRU) and CNN-RNN hybrid architecture, were explored to determine an optimal model. The comparative experimental results indicate the effectiveness of the CNN model for the classification of Chinese EMR data, which indicates that word order does not appear to have a useful effect on our Chinese EMRs. Furthermore, one-layer CNN performed best among all the classification applications. To conclude, the one-layer CNN model might contribute to the diagnosis of pediatric Chinese EMRs.

In this study, we only used EMR data and did not integrate medical images into the model. Therefore, future research will focus on how to integrate multiple types of medical information to improve the prediction effect for pediatric Chinese EMRs.

## Additional file

Additional file 1 Distribution of datasets with respect to four types of classification applications for pediatric Chinese EMRs. (PDF 142 kb)

## Abbreviations

CNN: Convolutional neural network
DMCNN: Multi-pooling CNN
EHR: Electronic health record
EMRs: Electronic medicine records
GRU: Gated recurrent unit
HAL: Hyperspace analog to language
HF: Heart failure
IHDPS: Intelligent heart disease prediction system
KB: Knowledge base
LSTM: Long short-term memory
MCCNN: Multi-column CNN
NER: Named entity recognition
NLP: Natural language processing
QA: Question and answer
RNN: Recurrent neural networks
