# Supplementary material for: Intelligent diagnosis with Chinese electronic medical records based on convolutional neural networks
Source: BMC Bioinformatics. 2019 Feb 1;20:62. doi: 10.1186/s12859-019-2617-8 (PMC6359854; doi:10.1186/s12859-019-2617-8)
Supplement: Supplementary file 1 — Distribution of datasets with respect to four types of classification applications for pediatric Chinese EMRs. (PDF 142 kb) [file 12859_2019_2617_MOESM1_ESM.pdf]

Distribution of datasets with respect to four types of classification applications for pediatric Chinese EMRs

| Number of diseases | English name of diseases | Chinese name of diseases | Number of samples |
|---|---|---|---|
| 7 | allergic rhinitis, bronchitis, acute bronchitis, respiratory disease, bronchial asthma, no critical, diarrhea, cough variant asthma | 变应性鼻炎； 支气管炎； 急性支气管炎； 呼吸道疾病； 支气管哮喘， 非危重； 腹泻； 咳嗽变异性哮喘 | 49,148 |
| 8 | **acute upper respiratory tract infection**, allergic rhinitis, bronchitis, acute bronchitis, respiratory disease, bronchial asthma, no critical, diarrhea, cough variant asthma | 急性上呼吸道感染； 变应性鼻炎； 支气管炎； 急性支气管炎； 呼吸道疾病； 支气管哮喘， 非危重； 腹泻； 咳嗽变异性哮喘 | 92,744 |
| 32 | *acute upper respiratory tract infection; allergic rhinitis; bronchitis; acute bronchitis; respiratory disease; bronchial asthma, no critical; diarrhea; cough variant asthma*; bronchopneumonia; acute asthmatic bronchitis; abdominal pain; enterovirus infection; fever; acute nasopharyngitis; cough; herpangina; acute tonsillitis; health examination; infantile enteritis; growth hormone deficiency; acute suppurative tonsillitis; acute sinusitis; gastroenteritis; acute gastroenteritis; urinary tract infection; asthmatic bronchitis; epilepsy; pneumonia; constipation; indigestion; acute lower respiratory tract infection; mycoplasma infection; | **急性上呼吸道感染; 变应性鼻炎; 支气管炎; 急性支气管炎; 呼吸道疾病; 支气管哮喘， 非危重; 腹泻; 咳嗽变异性哮喘**; 支气管肺炎; 急性喘息性支气管炎; 腹痛; 肠病毒感染; 发热; 急性鼻咽炎; 咳嗽; 疱疹性咽峡炎; 急性扁桃体炎; 健康查体; 小儿肠炎; 生长激素缺乏症; 急性化脓性扁桃体炎; 急性鼻窦炎; 胃肠炎; 急性胃肠炎; 泌尿道感染; 哮喘性支气管炎; 癫痫; 肺炎; 便秘; 消化不良; 急性下呼吸道感染; 支原体感染; | 132,637 |
| 63 | *acute upper respiratory tract infection; allergic rhinitis, bronchitis, acute bronchitis, respiratory disease, bronchial asthma, no critical, diarrhea, cough variant asthma; bronchopneumonia; acute asthmatic bronchitis; abdominal pain; enterovirus infection; fever; acute nasopharyngitis; cough; herpangina; acute tonsillitis; health examination; infantile enteritis; growth hormone deficiency; acute suppurative tonsillitis; acute sinusitis; gastroenteritis; acute gastroenteritis; urinary tract infection; asthmatic bronchitis; epilepsy; pneumonia; constipation; indigestion; acute lower respiratory tract infection; mycoplasma infection*; nausea and vomiting; idiopathic thrombocytopenia purpura(ITP); acute lymphoblastic leukemia; infantile diarrhea; gastritis; allergic purpura; gastrointestinal dysfunction; neonatal hyperbilirubinemia; hematuria; tic disorders; digestive system disease; upper respiratory tract hypersensitivity reaction; enuresis; neonatal jaundice; enteritis; mucocutaneous lymph node syndrome; renal allergic purpura; ulcerative stomatitis; routine examination of children's health; herpangina; chronic sinusitis; upper respiratory disease; stomatitis; right inguinal hernia; hyperthyroidism; anemia; helicobacter pylori infection; acute pharyngitis; left inguinal hernia; headache; acute laryngitis | **急性上呼吸道感染; 变应性鼻炎; 支气管炎; 急性支气管炎; 呼吸道疾病; 支气管哮喘， 非危重; 腹泻; 咳嗽变异性哮喘; 支气管肺炎; 急性喘息性支气管炎; 腹痛; 肠病毒感染; 发热; 急性鼻咽炎; 咳嗽; 疱疹性咽峡炎; 急性扁桃体炎; 健康查体; 小儿肠炎; 生长激素缺乏症; 急性化脓性扁桃体炎; 急性鼻窦炎; 胃肠炎; 急性胃肠炎; 泌尿道感染; 哮喘性支气管炎; 癫痫; 肺炎; 便秘; 消化不良; 急性下呼吸道感染; 支原体感染**; 恶心和呕吐; 血小板减少性紫癜; 急性淋巴细胞白血病; 婴儿腹泻; 胃炎; 过敏性紫癜; 胃肠功能紊乱; 新生儿高胆红素血症; 血尿; 抽动障碍; 消化系统疾病; 上呼吸道过敏反应; 遗尿; 新生儿黄疸; 肠炎; 粘膜皮肤淋巴结综合征[川崎病]; 肾型过敏性紫癜; 溃疡性口炎; 常规儿童健康检查; 咽峡炎; 慢性鼻窦炎; 上呼吸道疾病; 口腔炎; 右侧腹股沟斜疝; 甲状腺机能亢进症; 贫血; 幽门螺旋杆菌感染; 急性咽炎; 左侧腹股沟斜疝; 头痛; 急性喉炎; | 144,170 |
